# Treatment efficacy of low-dose 5-fluorouracil with ultrasound in mediating 5-fluorouracil-loaded microbubble cavitation in head and neck cancer

**DOI:** 10.1080/10717544.2022.2154410

**Published:** 2022-12-29

**Authors:** Ai-Ho Liao, Yu-An Lee, Dao-Lung Lin, Ho-Chiao Chuang, Jehng-Kang Wang, Ching-En Chang, Hsiang-Tzu Li, Ting-Yi Wu, Cheng-Ping Shih, Chih-Hung Wang, Yueng-Hsiang Chu

**Affiliations:** a Graduate Institute of Biomedical Engineering, National Taiwan University of Science and Technology, Taipei, Taiwan; b Department of Biomedical Engineering, National Defense Medical Center, Taipei, Taiwan; c Spirit Scientific Co., Ltd, Taiwan Branch (Cayman), New Taipei City, Taiwan; d Department of Mechanical Engineering, National Taipei University of Technology, Taipei, Taiwan; e Department of Biochemistry, National Defense Medical Center, Taipei, Taiwan; f Department of Otolaryngology–Head and Neck Surgery, Tri-Service General Hospital, National Defense Medical Center, Taipei, Taiwan; g Graduate Institute of Medical Sciences, National Defense Medical Center, Taipei, Taiwan

**Keywords:** Microbubbles, ultrasound, half-life, 5-fluorouracil, head nd neck cancer, cavitation

## Abstract

Over the past 50 years, 5-fluorouracil (5-FU) has played a critical role in the systemic chemotherapy of cancer patients. Bolus intravenous (IV) 5-FU infusion has been used due to the limitation of its extremely short half-life (10–15 min). This study used ultrasound (US) mediating 5-FU-loaded microbubbles (MBs) cavitation as a tool to increase local intratumoral 5-FU levels with a reduced dose of 5-FU (a single IV injection of 2.5 mg/kg instead of a single intraperitoneal injection of 25–200 mg/kg as used in previous studies in mice). The 5-FU-MBs were prepared with a 132 mg/mL albumin solution and a 0.30 mg/mL 5-FU solution. The diameters of the MBs and 5-FU-MBs were 1.24 ± 0.85 and 2.00 ± 0.53 µm (mean ± SEM), respectively, and the maximum loading efficiency of 5-FU on MBs was 19.04 ± 0.25%. In the *in vitro* study, the cell viabilities of 5-FU and 5-FU-MBs did not differ significantly, but compared with the 5-FU-MBs treatment-alone group, cell toxicity increased to 31% in the 5-FU-MBs + US group (*p* < 0.001). The biodistribution results indicated that the 5-FU levels of the tumors in small animals were significant higher for the 5-FU-MBs + US treatment than for either the 5-FU-MBs or 5-FU treatment with low 5-FU systemic treatment doses (2.5 mg/kg 5-FU IV). In small-animal treatment, 2.5 mg/kg 5-FU therapeutic IV doses injected into mice caused a more-significant reduction in tumor growth in the 5-FU-MBs + US group (65.9%) than in the control group after 34 days of treatment.

## GRAPHICAL ABSTRACT

**Figure:**
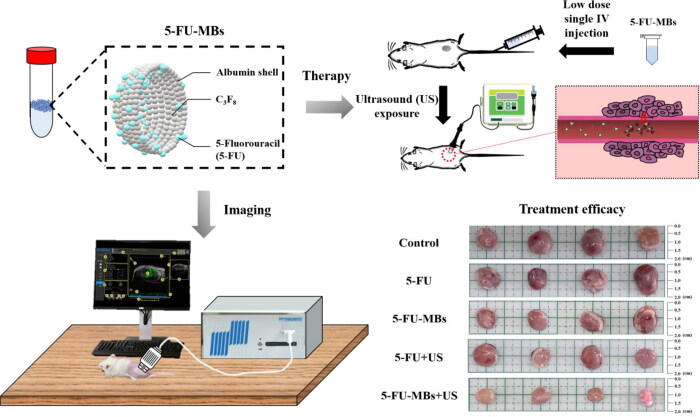

## 1. Introduction

The heterocyclic aromatic organic compound 5-fluorouracil (5-FU, chemical formula C_4_H_3_FN_2_O_2_), an analogue of uracil, a type of fluoropyrimidine, a fluorine organic compound, and an antimetabolite, has been widely used anticancer drug for gastric, breast, and head and neck cancers (Danneberg et al., 1958; Diasio & Harris, 1989; Grem, 2000). 5-FU is a first-line chemotherapeutic drug for colorectal cancer, which the main mechanism of action is related to thymidylate synthase inhibition, and leads to cytotoxicity and cell death (Grem, 2000). It can also enhance the therapeutic activity of other antineoplastic agents or modalities by synergizing with cisplatin and ionizing radiation (Grem, 2000). However, its clinical application is limited by the development of drug resistance after chemotherapy (Sethy & Nath Kundu, 2021). Intravenous (IV) bolus injections are severely limited by the extremely short half-life of 5-FU of 10–15 min, and so continuous IV 5-FU infusion over 46 hours has now become the standard fluoropyrimidine administration schedule (Lee et al., 2016; Draper et al., 2021). A recent clinical study indicated that cisplatin alone has fewer adverse events and better overall survival rates than cisplatin with 5-FU (Rades et al., 2016). In our present study, a new platform to inhibit the limitation of 5-FU was set up. 5-FU-loaded microbubbles (MBs) were created and combined with ultrasound (US) as a tool to increase the local intratumoral 5-FU level with a reduced 5-FU dose by using an IV bolus injection.

Folinic acid, a derivative of folic acid, is often combined with 5-FU and is used in the treatment of various cancers (Scaglione & Panzavolta, 2014). Previous researches found that increased folinic acid level potentiates the cytotoxic effects of 5-FU in the cancer cell (de Gramont et al., 2000). In clinical practice, receiving combined folinic acid and 5-FU treatment prolongs progression-free survival and improves response rates in patients with advanced, nonresectable colon adenocarcinoma. A novel pH-sensitive nanoparticulate drug-delivery vehicle was recently designed for oral use, which was capable of colon-specific release in colon cancer treatment (Ibrahim et al., 2022). On the other hand, it was more often used for IV 5-FU infusion as opposed to administering oral capecitabine (Ishikawa et al., 1998), tegafur-uracil (UFT) (Fujii et al., 1978) and tegafur-gimeracil-oteracil (S-1) (Shirasaka et al., 1996) because patients with head and neck cancer often suffer from dysphagia. In clinical practice, 5-FU is administered via IV infusion at a 7–12 mg/kg dose for 4 days for successive chemotherapy since its erratic absorption hampers bioavailability (Sharma et al., 2017). However, the limitations of 5-FU include its short biological half-life due to rapid metabolism (Diasio & Lu, 1994) and toxic side effects on bone marrow cells (Schilsky et al., 1998).

A modified systemic 5-FU chemotherapy protocol was assessed with the aim of decreasing hospitalization and/or treatment time without compromising outcomes, which could increase the life quality of patients with metastatic squamous cell carcinoma of the head and neck (SCCHN) (Meirovitz et al., 2022). More recently, a new strategy using the immune checkpoint inhibitor pembrolizumab alone or in combination with 5-FU and platinum has become an appropriate first-line treatment for patients with recurrent or metastatic head and neck cancer (Falco et al., 2022). 5-FU is accessible to the relevant tissues in conjugation with the transport protein serum albumin (Chinnathambi et al., 2016). It has strong binding affinity to human serum albumin (HSA), and binds to the lower region of the proximal site in subdomain IB and interacts with alpha helices H7, H8, and H9. Moreover, the uracil ring of 5-FU has a π − π interaction with Phe149 and His146, a hydrogen bond with Tyr138, and many hydrophobic interactions with HSA (Chinnathambi et al., 2016). 5-FU was therefore conjugated to recombinant HSA nanoparticles to improve its pharmacokinetic and therapeutic profiles (Sharma et al., 2017). Our previous studies indicate that head and neck cancer is the most-promising target for chemotherapy aided by combining US and MBs (Chen et al., 2018). The use of cisplatin-loaded HSA MBs together with US enhanced the antitumor effects in areas that received localized US sonication while reducing the toxicity of the kidney and liver, and improving the distribution and treatment efficacy. In the present study, a 5-FU conjugated HSA complex was produced to form injectable MBs, and was combined with US to prolong its half-life, shorten the treatment procedure, and improve the local treatment effects.

## 2. Materials and methods

### 2.1. Preparation and characterization of 5-FU-loaded albumin-shelled MBs

**Figure 1.**
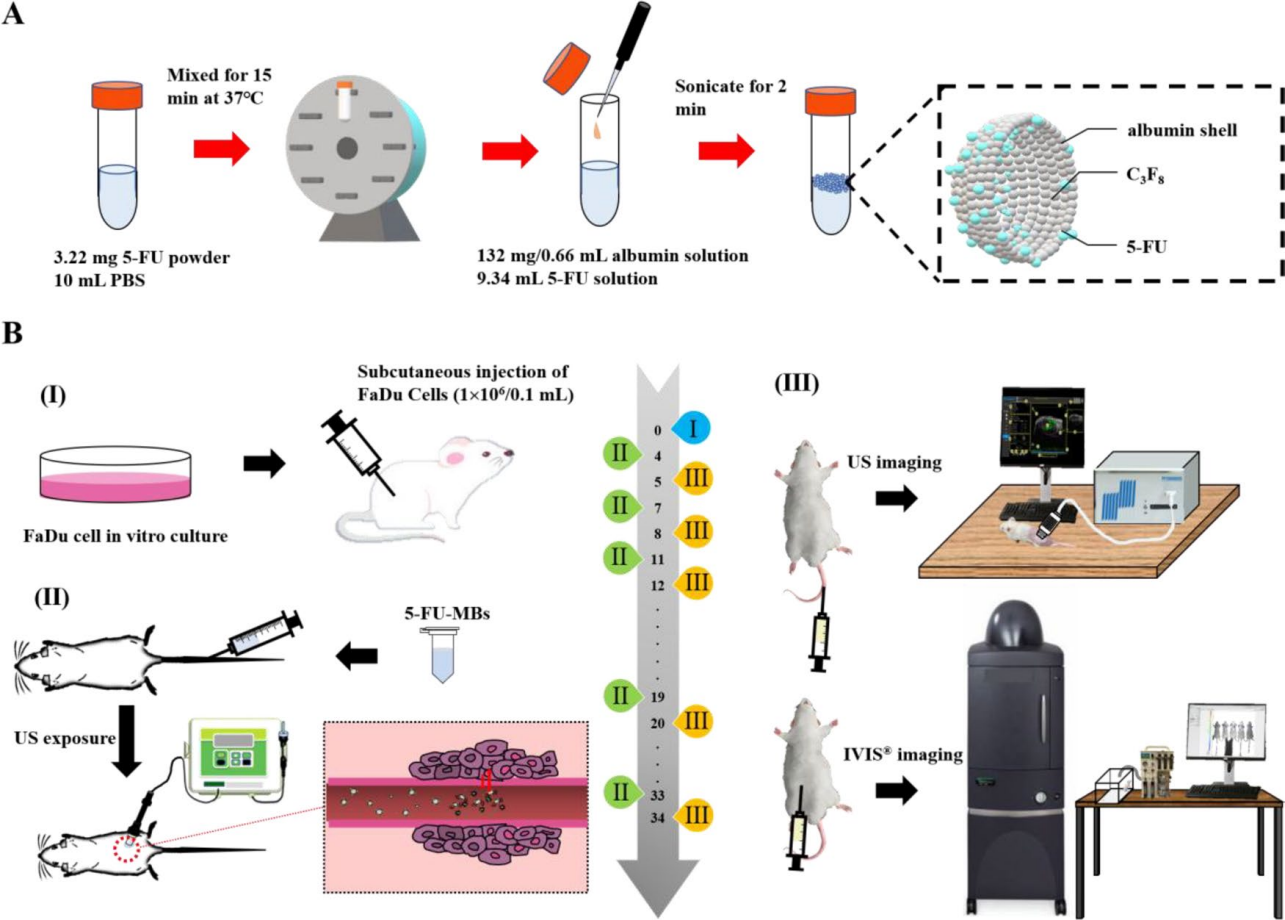
Schematics of (A) the preparation process of 5-FU-MBs and (B) the animal experiment design.

As illustrated in Figure 1(A), the method used to prepare the 5-FU-MBs was modified as follows: 3 mg of 5-FU powder (C_4_H_3_FN_2_O_2_, molecular weight [MW] = 130.08, ≥99.9% [high-performance liquid chromatography, HPLC], Sigma-Aldrich, St. Louis, IL, USA) in 9.34 mL of phosphate-buffered saline (PBS) was mixed at 37 °C in a vortex mixer (65 rpm) for 15 min, and 132 mg/0.66 mL HSA (Octapharma, Vienna, Austria) was then added followed by incubation on ice for 20 min. The sample was then subjected to 2 min of sonication with perfluorocarbon (C_3_F_8_) gas using a sonicator (Branson Ultrasonics, Danbury, CT, USA). Only 6 mL of 5-FU-MBs could be isolated after the sonication process. The sample was then centrifuged in 1 mL of PBS at 1,200 rpm (110 xg) for 2 min (Thermo Fisher Scientific, Bremen, Germany). The lower layer was removed to eliminate the free (unbound) 5-FU, 1 mL of PBS was added, and the sample was then stored at 4 °C. The size distribution of 5-FU-MBs in the solution was measured using dynamic light scattering (SZ-100 Nanoparticle Analyzer, Horiba, Kyoto, Japan), and the number of MBs was measured using the MultiSizer III device (Beckman Coulter, Fullerton, CA, USA) with a 30-μm aperture probe with a measurement range of 0.6–20 μm. The morphology of the 5-FU-MBs was characterized by filtering 40-fold-diluted 5-FU-MBs using a 5-µm syringe filter (Sartorius, Goettingen, Germany), and 5 µL of MB and 5-FU-MB samples were then mounted on copper stubs with double-sided carbon adhesive tape and then coated with platinum (achieved at 0.1 nm/s and 30 mA for 60 s) using the JFC-1300 automatic sputter coater (JEOL, Tokyo, Japan). The morphologies of the MBs and 5-FU-MBs were characterized using high-resolution field-emission scanning electron microscopy (FESEM; JSM-6500F, JEOL, Tokyo, Japan) at a 15-kV accelerating voltage. Before measuring the loading efficiency of 5-FU on 5-FU-MBs, the 5-FU-MBs were sonicated with US energy at a 3 W/cm^2^ power density (ST2000V, Nepa Gene, Ichikawa, Japan; acoustic pressure = 0.266 MPa, I_SPTA_=0.655 W/cm^2^) for 1 min to destroy the 5-FU-MBs. The concentrations before and after MB destruction were measured using the MultiSizer III device (Beckman Coulter), which revealed that the sonication process destroyed 95% of the MBs. 5-FU and 5-FU-MB fragments were determined at various concentrations in the supernatant using the Lambda 40 UV-visual spectrophotometer (PerkinElmer, Bridgeville, PA, USA). The HSA or 5-FU calibration curves served as the standard curves against which the absorption peaks and corresponding HSA or 5-FU concentrations in the samples were measured. The results indicate that HSA absorbs light at 280 nm, whereas 5-FU and 5-FU-MBs absorb light at 266 nm.

### 2.2. In vitro release study

In the *in vitro* release study, 3 mL of a 5-FU-MB suspension (of the original concentration after production) was loaded into a dialysis bag (MW cutoff = 12,000–14,000) and was dialyzed against the 100 mL of PBS release media at pH values of 5.0 and 7.4 and at 36.5–37.5 °C, and stirred using a magnetic bar at 600 rpm. After 0.5 hours, the 1-MHz unfocused US transducer of the ST2000V sonoporation system (Nepa Gene) positioned 3 mm from the top of the dialysis bag under the liquid level provided sonication at a 3 W/cm^2^ power density for 5 min. After 0.1, 0.2, 0.3, 0.4, 0.5, 1, 2, 3, 4, 5, and 6 hours, 1 mL samples were taken from the release medium and the same volume of PBS was added each time to replace the release medium. These samples were analyzed using a UV-visual spectrophotometer (Lambda 40, PerkinElmer). The drug release profile of 5-FU was examined as a control.

### 2.3. Optimization of in vitro US parameters for MB destruction

Our previous study demonstrated that drug delivery enhancement is related to the destruction efficacy of MBs (Liao et al., 2020). In this experiment, *in vitro* US parameters for MB destruction were optimized to not affect cell survival. A 24-well plate was filled with about 4 mL of MBs (1.0 × 10^8^ MBs/mL) in each well while ensuring that no air bubbles were trapped when the cover was placed. An US instrument equipped with a 10-mm diameter probe operating at a 1 MHz center frequency and 50% duty cycle was used for sonication (ST2000V, Nepa Gene). The probe was placed directly on the 24-well cover, with gel used as an US coupling agent. The US parameters for *in vitro* MB destruction were established by investigating the effects of US sonication at 1 W/cm^2^ for 30 s two, four, or six times, or for 1 min once.

After each US sonication, the MB solution in each well was diluted ten times and then imaged using an US animal imaging system (Prospect, S-Sharp Corporation, Taipei, Taiwan). Images were processed using custom MATLAB programs (The MathWorks, Natick, MA, USA) to evaluate the destruction efficiency by calculating the difference in the gradient strength on the MB images between before and after US sonication. The *in vitro* effects of US-mediated MB destruction on the FaDu cell line (human pharyngeal squamous carcinoma cell line, American Type Culture Collection, HTB-43) were evaluated on the 24-well plate at 1 × 10^5^ cells/well overnight. Each well was filled with about 4 mL of MBs (1.0 × 10^8^ MBs/mL) the next day, followed by US sonication as described above. After US sonication, the MB solution was replaced with a culture medium (minimum essential medium, MEM, Thermo Fisher Scientific, Waltham, MA, USA) and the cells were allowed to grow for 48 hours. The alamarBlue™ reagent assay was then used to assess cell viability.

### 2.4. Cell viability assay and in vitro cytotoxicity of 5-FU-MBs in the FaDu cell line

The cell culture medium was replaced with a 500 μL/well alamarBlue™ Cell Viability Reagent (Roche, Mannheim, Germany) and the cells were incubated for another hour. alamarBlue™ assays incorporate a fluorometric/colorimetric growth indicator based on metabolic activity detection. As cells grow while being tested, innate metabolic activity results in a chemical reduction of the alamarBlue™ reagent (Sephra, 2012). Continued growth maintains a reduced environment while inhibition of growth maintains an oxidized environment, and growth-related reduction causes the fluorometric/colorimetric indicator to change from its oxidized (nonfluorescent, blue) to reduced its (fluorescent, red) form. The amount of the reduced form was quantified by measuring fluorescence signals at a 530–560 nm excitation wavelength and a 590-nm emission wavelength using an ELISA (enzyme-linked immunosorbent assay) Reader (Synergy H4 Hybrid Reader; BioTek Instruments, Winooski, VT, USA). Wells without cells were used as the blanks. The percentage cell viability was calculated using the following formula: cell viability = cells (sample)/cells (control) × 100.

FaDu cells (0.5 mL) with 10% FBS at 1 × 10^5^ cells/well were contained in the 24-well plates and were cultured in humidified air with 5% CO_2_ at 37 °C overnight. They were then divided into five groups (*n* = 6 per group) for incubation with different combinations of 5-FU, MBs, and 5-FU-MBs for 48 hours with or without US sonication (1 W/cm^2^, 30 s six times) as follows: group 1, Control; group 2, MBs only (1.0 × 10^8^ MBs/mL); group 3, 5-FU only (0.5, 1.25, and 2.5 μM); group 4, 5-FU (0.5, 1.25, and 2.5 μM) with MBs (9.9 × 10^7^ MBs/mL); and group 5, 5-FU-MBs (5-FU loading content: 0.5, 1.25, and 2.5 μM in 9.9 × 10^7^ MBs/mL). After 48 hours of incubation, the cell viability was assessed using the alamarBlue™ assay.

### 2.5. High-frequency US imaging to determine in vivo MB destruction

The optimal parameters for maximizing *in vivo* sonication efficiency differ from those for *in vitro*, and so the adherent MBs in xenograft tumors in mice and the subsequent US-mediated MB destruction were monitored by high-frequency US imaging using a commercial small-animal US imaging system (Prospect, S-Sharp Corporation) with agarose phantoms (Liao et al., 2012). The US images were obtained using a transducer with a 40-MHz central frequency and axial and lateral resolutions of 30 and 60 µm, respectively. The axial and lateral fields of view were both 20 mm. Real-time US B-mode imaging was performed at a 20-Hz frame rate (corresponding to a 50-ms temporal resolution). Two-dimensional image planes were acquired with optimized gain and time-gain compensation settings, which were kept constant throughout the experiments under the axial and lateral fields of view.

The mice were anesthetized with 2% isoflurane before the contrast-enhanced *in vivo* tumor imaging experiments, and the hair covering the tumor region was removed. The transducer of the high-frequency US imaging device was fixed onto a railing system with the acoustic focus centered at the level of the subcutaneous tumor xenografts, and the imaging plane was aligned at the center of the tumor. After injecting 5-FU-MBs into the lateral tail vein, the sonoporation gene transfection system probe (1 MHz, 50% duty cycle, I_SPTA_=0.655 W/cm^2^; ST2000V, Nepa Gene) was applied at 3 W/cm^2^ for 30 s. Images representing the 5-FU-MBs in the tumor were displayed as green overlays on the B-mode anatomic images.

### 2.6. Small-animal tumor treatment experimental design

Figure 1(B) illustrates the design of the animal study. The SCCHN tumor-bearing mouse model was established in severe combined immunodeficiency specific pathogen-free (SPF) SCID (CB17/lcr-*Prkdc*^scid^/lcrlcoCrlBltw) mice after inducing general anesthesia using isoflurane inhalation (2% in oxygen). On day 0, the mice were subcutaneously injected with 0.1 mL of a FaDu-fLuc/GFP cell suspension (1 × 10^6^ cells/mouse) into the right flank region using a 1-mL Monoject Tuberculin Safety Syringe with a 25 gauge needle (Figure 1BI). Because of the differences in tumor sizes, mice with similar tumor sizes were equally distributed and divided into five groups of five animals each: group I, PBS Control (0.2 mL); group II, 5-FU (0.5 mg 5-FU, 0.2 mL; therapeutic dose of 2.5 mg/kg); group III, 5-FU-MBs (0.5 mg 5-FU, 1.99 × 10^7^ HSA MBs/0.2 mL); group IV, 5-FU + US (0.5 mg 5-FU; US sonication at 3 W/cm^2^, 30 s); and group V, 5-FU-MBs + US (0.5 mg 5-FU, 1.99 × 10^7^ 5-FU-loaded HSA MBs/0.2 mL; US sonication at 3 W/cm^2^, 30 s).

PBS, 5-FU, MBs, and 5-FU-MBs were administered via IV injection at a volume of 0.2 mL in the tail vein on days 4, 7, 11, 15, 19, 23, 27, 30, and 34 (a total of nine injections). Following each injection, the US probe was immediately applied to the tumors of mice from groups IV and V for 30 s (Figure 1BII). Before each chemotherapy session, tumor volume was assessed using the small-animal US imaging and bioluminescence image (BLI) systems (Perkin Elmer) (Figure 1BIII). Tumor xenograft formation and size were monitored and evaluated on day 5 using the BLI system via an intraperitoneal injection of 150 mg/kg D-luciferin (Biosynth, Staad, Switzerland). After a 10-min distribution period, the tumors were quantified by measuring the total photon count per second. A digital grayscale animal image was acquired, and the pseudocolor image was overlaid to represent the spatial distribution of the detected photon counts that emerged from active luciferase within the tumor. The luminescence signal intensity was quantified as the radiance. The tumor volumes were quantified by measuring the total photon count per second within the region of interest.

### 2.7. Biodistributions of 5-FU, 5-FU-MBs, and 5-FU-MBs + US

After the experimental treatments, the xenograft tumor-bearing mice in each group were injected intraperitoneally with barbiturate (150 mg/kg) and sacrificed by cervical dislocation. Subsequently, their livers, blood, kidneys, and tumors were excised, immediately frozen in liquid nitrogen, and stored at −80 °C until the analysis. Plasma and tissue samples after weighing were homogenized in 2 mL of distilled ice water with a probe-type sonicator in operational mode with a 300-W pulse (3 s on and 2 s off) so that the temperature remained lower than 25 °C. The homogenate was then centrifuged at 10,000 rpm (11,180 xg) for 10 min. The supernatant was then collected and 200 μL of it was deproteinized using methanol and then ultrafiltrated using a Vivaspin 500 ultrafiltration tube (membrane with an MW cutoff of 10,000), with 500 μL of the diluted supernatant ultrafiltrated at 5,000 rpm (2795 xg) for 10 min at room temperature, and 200 μL of the obtained ultrafiltrate used for HPLC (1100, Agilent Technologies, Santa Clara, CA, USA). For HPLC, the mobile phase comprised a mixture of 5 mM phosphate buffer (pH 5)-methanol (95:5, v/v) that was degassed by sonication and filtered through a 0.22-mm Millipore membrane filter before use (Wang et al., 2011). The 20-μL injection volume of the sample was pumped through an SB-806MHQ-C18 column at a 0.5 mL/min flow rate (300 mm × 8.0 mm; Shodex, Showa Denko America, New York, NY, USA) at room temperature. 5-FU was detected on a UV detector (UV-2075, Jasco, Tokyo, Japan) at 265 nm.

### 2.8. Sample preparation for histopathological examinations

The xenograft tumor tissue, liver, and kidney excised from each sacrificed mouse in each group were fixed in a 10% formalin solution and processed for paraffin embedding. The paraffin*-*embedded tissue cassettes were mounted onto a rotary microtome, sectioned to an approximate thickness of 5 µm, and stained with hematoxylin and eosin (H&E). The sections were analyzed using a fluorescence microscope (BX50, Olympus, Tokyo, Japan) equipped with a digital camera (DP74, Olympus). The digital photomicrographs were processed using Olympus cellSens Standard software (version 1.17).

### 2.9. Statistical analysis

The obtained data were statistically analyzed using two-tailed Student’s *t*-tests. Groups were compared using one-way ANOVA followed by Tukey’s multiple-comparisons test. A probability value of *p* < 0.05 was considered indicative of a significant difference.

### 2.10. Study approval

This animal study was approved by the Institutional Animal Care and Use Committee of the National Defense Medical Center in Taipei, Taiwan. Animals were cared for in compliance with institutional guidelines and regulations (no. IACUC-19-065). SCID mice (female, aged 7–8 weeks) were obtained from BioLASCO (Taipei, Taiwan) and were subsequently housed at the Laboratory Animal Center of the National Defense Medical Center (Taipei, Taiwan) under SPF conditions, were used for experiments, and then cared for in accordance with institutionally approved protocols.

## 3. Results

### 3.1. 5-Fu-MBs characterization and 5-FU loading efficiency in 5-FU-MBs

**Figure 2.**
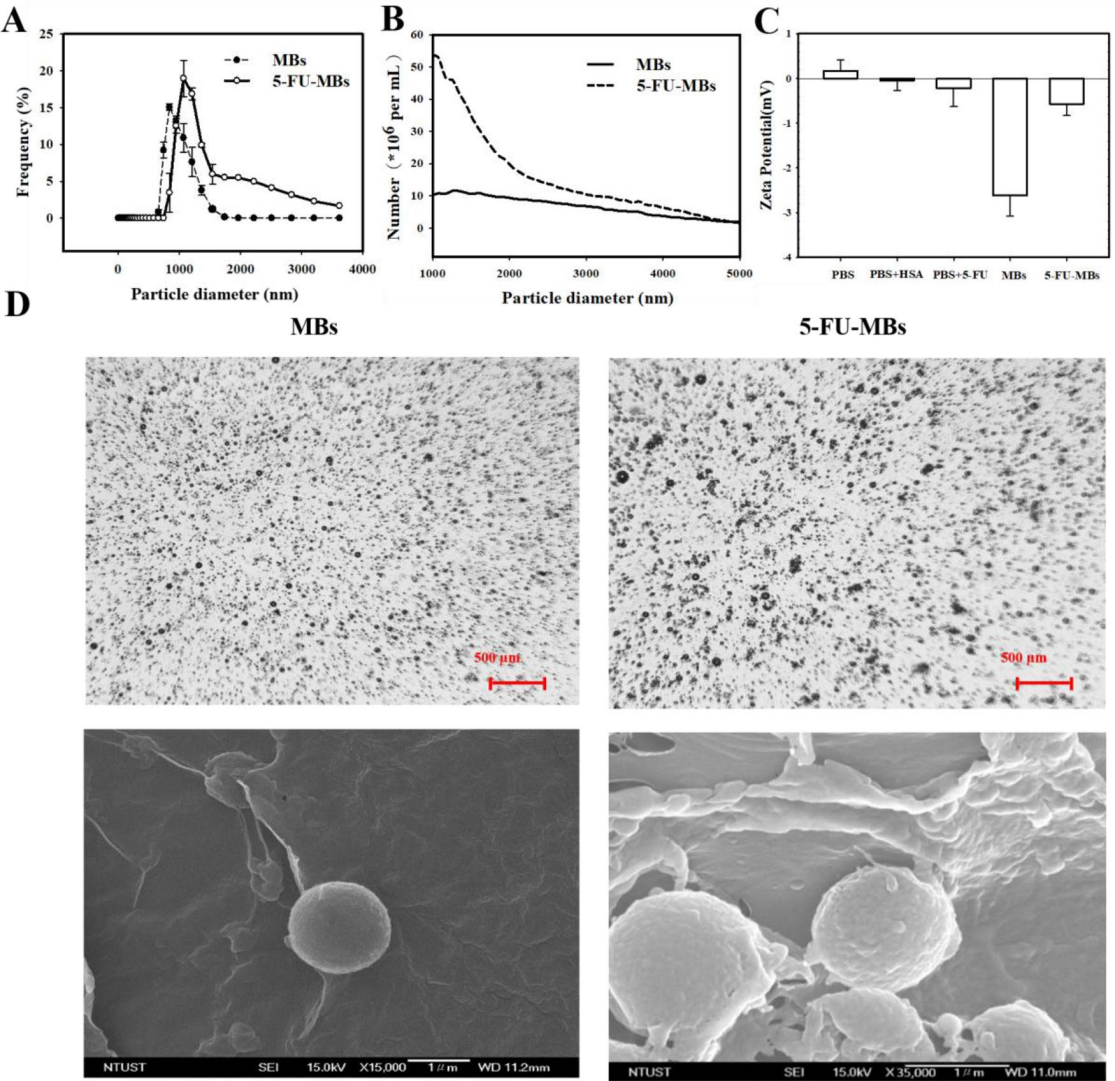
Light quantification and scanned electron microscopy images of unloaded MBs and 5-FU-MBs. (A) Frequency-dependent and (B) number-dependent size distributions of various MBs. Data are mean ± and SEM values (*n* = 5). (C) The zeta potentials of PBS, HSA in PBS, 5-FU in PBS, MBs in PBS, and 5-FU-MBs in PBS. (D) Representative light microscopy and scanning electron microscopy images of MBs and 5-FU-MBs.

The diameters of the HSA-shelled MBs and 5-FU-loaded HSA MBs were 1.24 ± 0.82 and 2.00 ± 0.53 µm (mean ± SEM), respectively (Figure 2A and Table 1); the corresponding of MB and 5-FU-MB concentrations were 7.44 ± 0.56 × 10^8^/mL and 9.97 ± 1.23 × 10^8^/mL (*n* = 6) (Figure 2B). The formal charge of 5-FU is close to zero, and the zeta potential of 5-FU-MBs in PBS (–0.57 ± 0.26 mV) was significantly higher than that of MBs in PBS (–2.62 ± 0.46 mV) (*p* = 0.003), but did not differ from that of the 5-FU in PBS (–0.22 ± 0.40 mV) (*p* = 0.15, *n* = 15) (Figure 2C). Figure 2(D) shows that the distribution density of 5-FU-MBs under a bright-field microscope was slightly lower than that of MBs, and that the particle size was not uniform. Since the drug was embedded and wound on a spherical shell, the high-resolution FESEM observation results indicated that the surface was relatively uneven, and the particle size of 5-FU-MBs also increased.

**Table 1.** Comparison of the diameter, number, and 5-FU loading efficiencies of albumin-shelled MBs and 5-FU-MBs. Data are mean ± SEM values.

Albumin concentration (mg/mL)	5-FU concentration (mg/10 mL)	Microbubble concentration (×108/mL)	Particle diameter (μm)	Encapsulation efficiency (%)
132	N/A	7.44 ± 0.56	1.24 ± 0.82	N/A
132	3.00	9.97 ± 1.23	2.00 ± 0.53	19.04 ± 0.24

**Figure 3.**
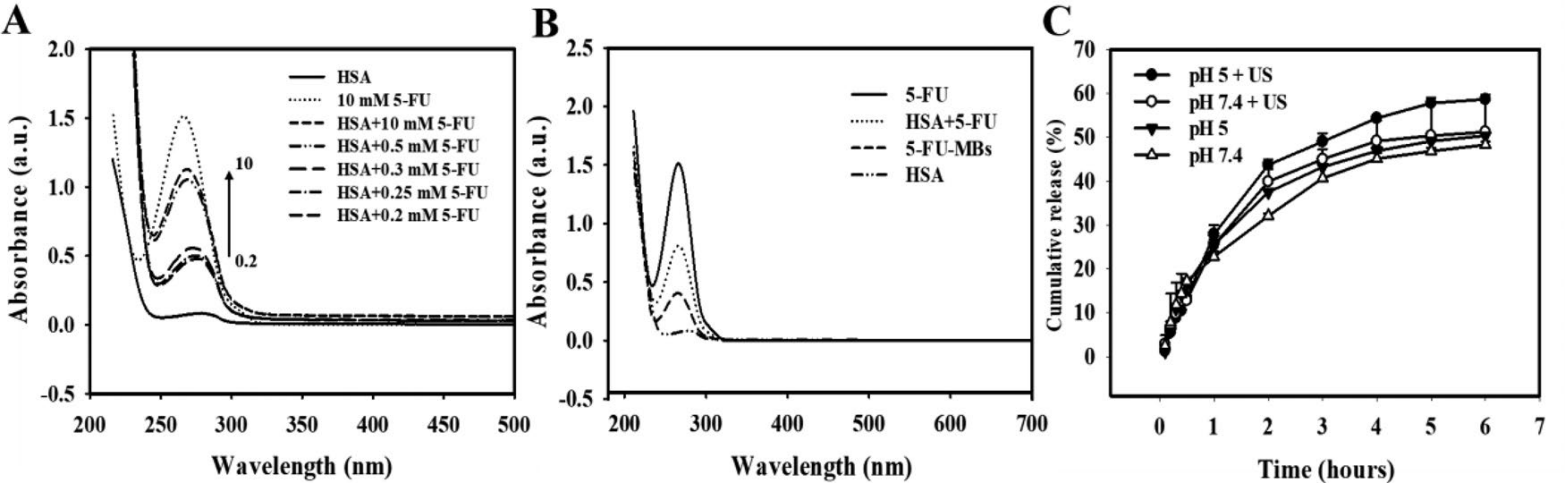
(A) UV absorption spectra of HSA according to 5-FU concentration in the HSA and PBS buffer solutions. (B) Absorbance spectra of HSA, HSA + 5-FU, 5-FU-MBs after US sonication (destruction), and HSA. (C) Comparative cumulative drug release of 5-FU at 6 hours after 5-FU-MBs administration with and without US sonication in PBS at pH 5 and 7.4.

The UV-visual absorption characterization in Figure 3(A) indicates the complex formed between 5-FU and proteins, and that the absorption spectra of the HSA–5-FU complex in the presence of FU increased with the 5-FU concentration. It was further observed that there was a blue shift in the maximum peak position of the HSA-5-FU complex, indicating a strong interaction between HSA and 5-FU. It can be observed in Figure 3(B) that there was a change in 5-FU-MBs absorbance and that some interaction changes occurred between HSA and 5-FU after the MB manufacturing process. The variation of 5-FU absorbance contrasted with the fixed HSA concentration (132 mg/mL, which was the optimal HSA concentration for manufacturing MBs), which had a steady absorbance at 3.00 mg/10 mL HSA. Analysis of the encapsulation efficiency of the 5-FU coated on the MB shells indicated a maximum loading efficiency for 5-FU-MBs of 19.04 ± 0.24% (Table 1). These 5-FU-MBs were used for the subsequent experiments involving *in vitro* 5-FU cytotoxicity, and in *in vivo* animal treatments.

### 3.2. In vitro 5-FU release from 5-FU-MBs

Figure 3(C) shows the percentage of cumulative 5-FU release after 6 hours in the 5-FU-MBs group in PBS for pH values of 5 and 7.4. At pH 5, 37.5% of the free 5-FU had diffused through the dialysis membrane relative to the control over the first 2 hours, and this increased to 43.7% with US sonication; at pH 7.4 these values were 32.1% and 39.9%, respectively. Without US sonication, the proportion of the free drug suspension released across the dialysis membrane was reduced to only 46.9% at pH 5 and 45.1% at pH 7.4 after 4 hours. With US sonication, the *in vitro* 5-FU release was rapid during the first 4 hours, reaching 54.3.8% at pH 5 and 49.1% at pH 7.4, followed by a slower but sustained 5-FU release from the 5-FU-MBs to just over 58.6% and 51.2%, respectively, at 6 hours. These results indicate that applying US energy enhanced drug release by 2.5–8.9%, and also that pH affects the efficiency of 5-FU release from 5-FU-MBs.

### 3.3. Us and MB optimization on in vitro 5-FU cytotoxicity

**Figure 4.**
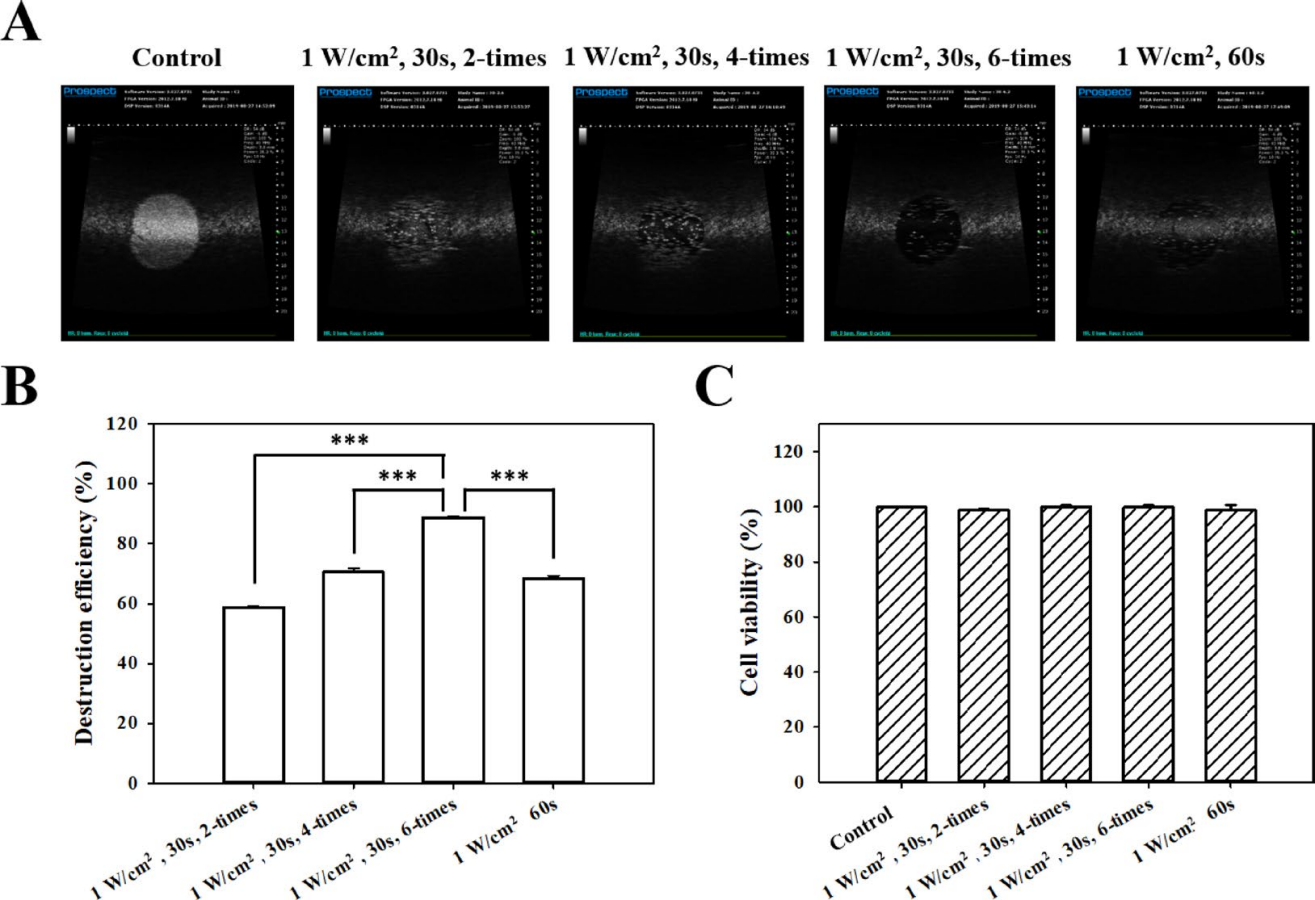
(A) *In vitro* high-frequency US images from MBs, and (B) corresponding image intensities in various groups of acoustic exposure settings. ***p < 0.001. (C) Percentage of cell viability evaluated using the alamarBlue™ assay on FaDu cells 48 hours after different acoustic exposure settings (*p* > 0.05).

High-frequency US images of the MBs groups with and without US sonication at 1 W/cm^2^ in a sonication-time–dependent manner are shown in Figure 4(A), and the destruction efficiencies are quantified in Figure 4(B). The destruction efficiencies of HSA MBs at exposure times of 30 s × two, 30 s × four, 30 s × six, and 60 s × one were 58.8%, 70.6%, 88.8%, and 68.4%, respectively. The optimal US sonication parameters for MB destruction at an *in vitro* exposure time of 30 s × six were significantly higher than for other US sonication parameters (*p* < 0.001). Figure 4(C) shows that six cycles of 1-W/cm^2^ US sonication for 30 s did not affect the viability of FaDu cells in culture (*p* > 0.05). Six cycles of US sonication for 30 s was consequently used for subsequent *in vitro* experiments.

### 3.4. 5-Fu reduces FaDu-cell viability in a concentration-dependent manner

**Figure 5.**
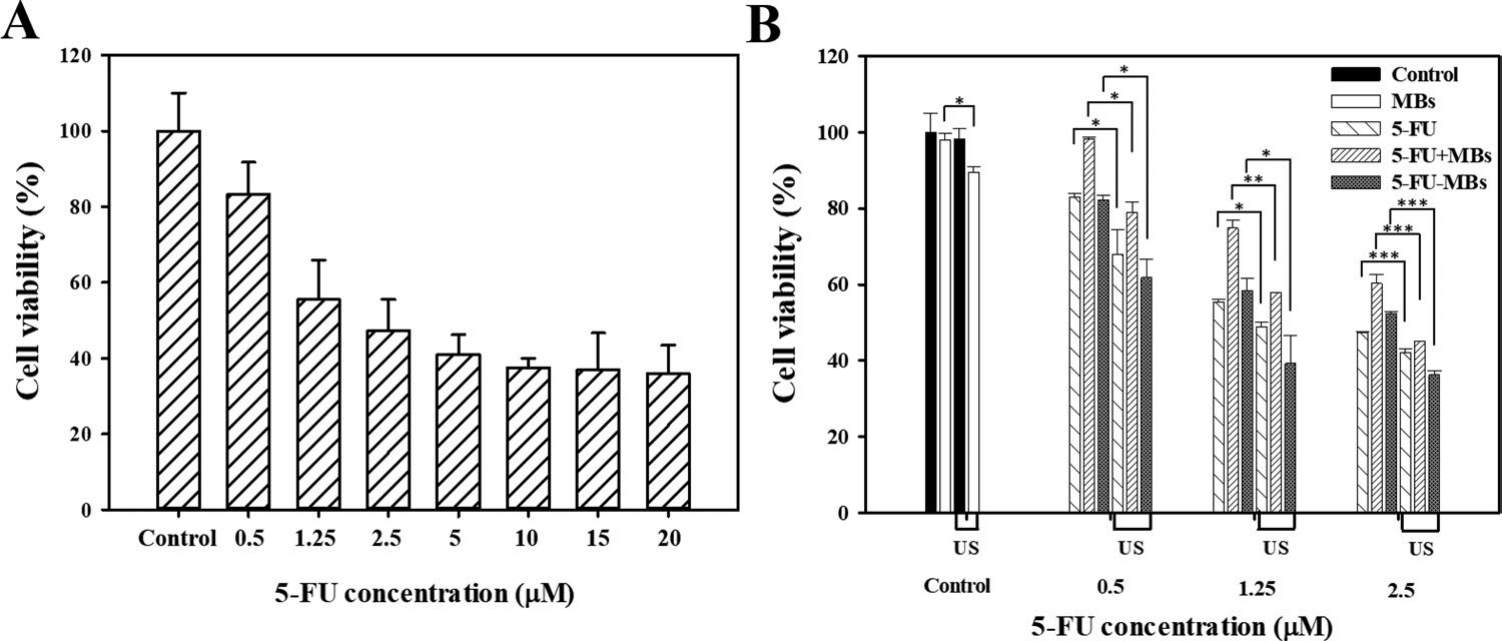
Effects of (A) 5-FU and (B) 5-FU only, 5-FU + MB, and 5-FU-MB treatments with or without US sonication on the viability of FaDu cells. The cells were treated with 5-FU at 0.5–20 µM and US + 5-FU at 0.5–2.5 µM for 48 hours, and then examined using the alamarBlue™ assay. Data are percentages relative to controls presented as mean and SEM values (*n* = 5). *p < 0.05, **p < 0.01, ***p < 0.001.

**Figure 6.**
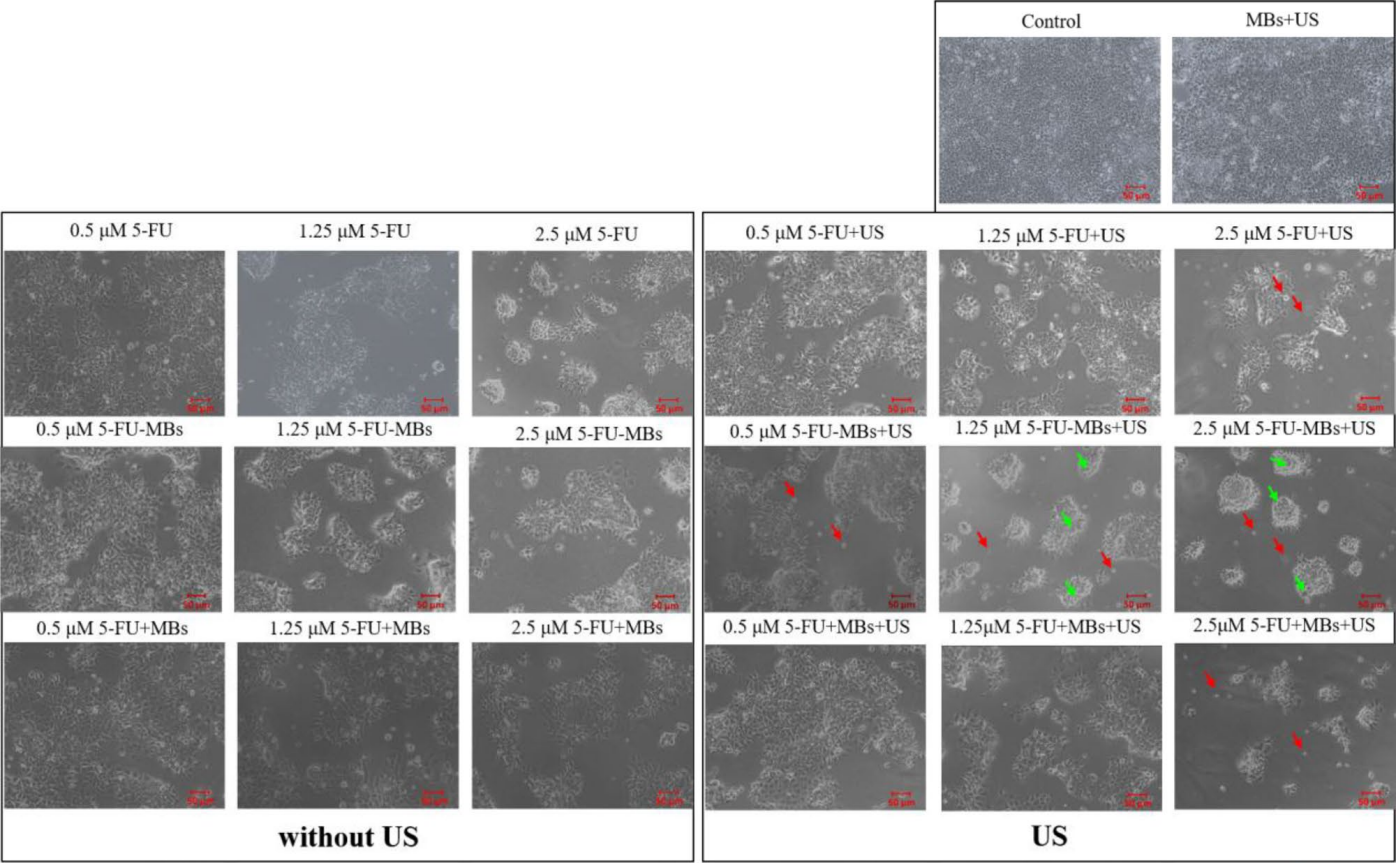
Light-microscopy photographs showing the effects of the control, 5-FU (0.5, 1.25, and 2.5 µM), 5-FU-MB, and 5-FU + MB treatments with or without US sonication. Green arrows show cells that became rounded and shrunken; red arrows indicate suspensions of dead cells.

Evaluating the effect of 5-FU on the viability of human FaDu pharyngeal squamous carcinoma cells indicated that 5-FU treatment from 0.5 to 20 µM for 48 hours decreased the cell viability from 82.97% to 35.89%, respectively (Figures 5A and 6). For FaDu cells treated with 5-FU, the IC50 value (half-maximum inhibitory concentration, corresponding to the concentration that reduces cell viability by 50%) was found to be 2.3 µM. The cells treated with 5-FU at 0.5, 1.25, and 2.5 µM, which individually were not higher than (or close to) the IC50 value, demonstrated the additive effects.

### 3.5. Influence of MBs, 5-FU-MBs, and US on cell viability for in vitro 5-FU cytotoxicity

Figures 5(B) and 6 show the viability of FaDu cells treated with 5-FU at different concentrations and when combined with MBs and US. The cell viability differed significantly between MBs (98.06 ± 1.76%) and US plus MBs (89.45 ± 1.42%) (*p <* 0.05). At a low concentration (0.5 μM) of 5-FU, the cell viabilities of 5-FU (83.00 ± 0.80%) and 5-FU-MBs (81.67 ± 1.18%) did not differ significantly, but the cytotoxicity was reduced after adding MBs to 5-FU (cell viability of 5-FU + MBs: 98.23 ± 0.58%). At various 5-FU concentrations (0.5, 1.25, and 2.5 μM), it was observed that the cytotoxicities in the 5-FU and 5-FU-MBs groups were similar. After US sonication, cytotoxicity was significantly higher in the 5-FU-MBs group than in the 5-FU group (*p <* 0.05). Moreover, the cell viability decreased significantly for 2.5 µM 5-FU-MBs + US (36.24 ± 1.04%), suggesting that combining 5-FU-MBs with US enhanced 5-FU cytotoxicity. Figure 6 shows that after 5-FU treatment or US sonication, the FaDu cells lost their original shape after 48 hours of treatment. The FaDu cells did not maintain their polygonal or elongated spindle-shape morphology, and some suspension cells (dead cells) were identified in both the 1.25 and 2.5 μM 5-FU-MBs + US groups.

### 3.6. Influence of MBs and US on the in vivo systemic 5-FU uptake

**Figure 7.**
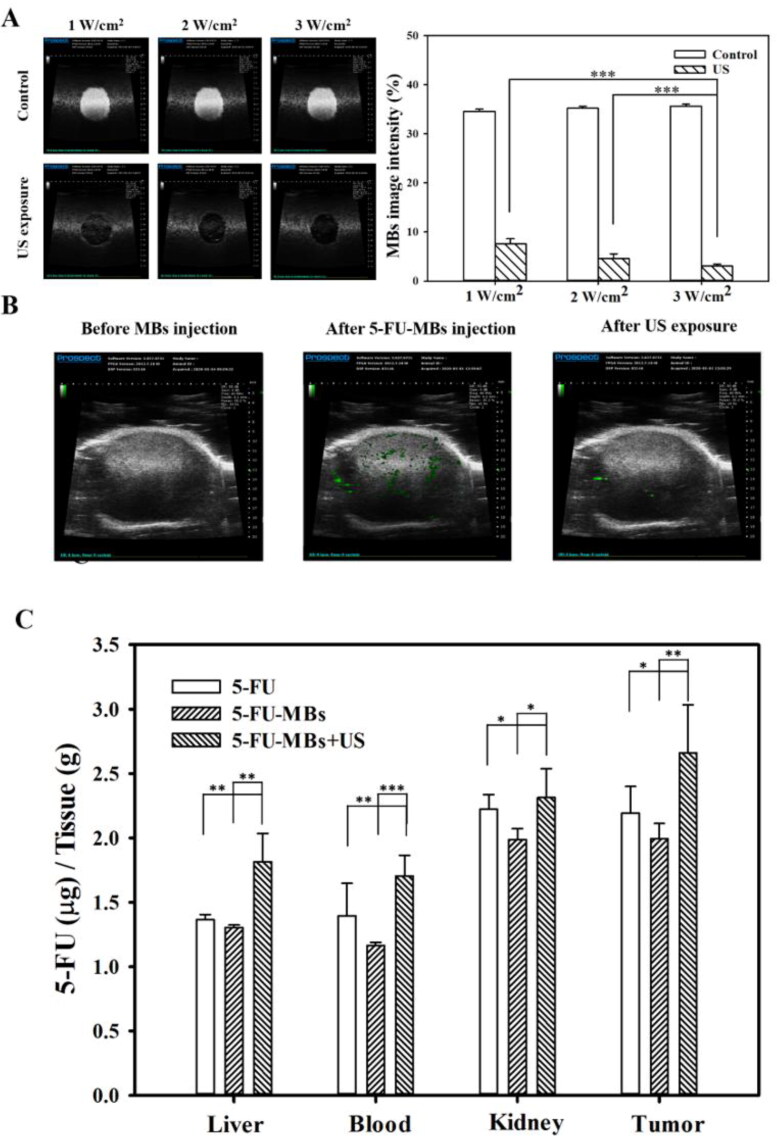
Influences of MBs and US on *in vivo* 5-FU accumulation. (A) High-frequency US images of albumin-shelled MBs in tissue-mimicking agarose phantoms before (control) and after sonication at 1, 2, and 3 W/cm^2^ for 30 s. The image intensities of MBs are quantified. (B) A series of high-frequency US images that focus on tumor lesions showing the presence of MBs (green) within the tumor after their administration via the mouse tail vein, and MB disappearance after 30 s of therapeutic US sonication. (C) 5-FU levels in vital organs were determined 10 min after a single IV administration of chemotherapy using the HPLC quantitative method. Data are mean and SEM values (*n* = 5). *p < 0.05, **p < 0.01, ***p < 0.001.

In Figure 7(A), the *in vivo* US mediated MBs cavitation (USMB) parameters were optimized by testing different acoustic power intensity settings in tissue-mimicking agarose phantoms. The destruction efficiencies of 5-FU-MBs at acoustic intensities of 1, 2, and 3 W/cm^2^ for 30 s were 78.48 ± 1.01%, 86.96 ± 0.94%, and 91.37 ± 0.42%, respectively. The US power and exposure duration were therefore set at 3 W/cm^2^ and 30 s, respectively, for all subsequent *in vivo* experiments. Figure 7(B) shows the *in vivo* high-frequency US tumor images demonstrating the real-time appearance of 5-FU-MBs within the tumor after tail vein MB injection. Destruction of 5-FU-MBs at the tumor site was then achieved through US sonication at 3 W/cm^2^, and the 5-FU-MBs subsequently disappeared after US sonication for 30 s. These images indicate that IV-injected 5-FU-MBs circulated to the tumor lesions, where they could be destroyed by US sonication, thereby releasing the 5-FU from the 5-FU-MBs.

The biodistribution of the prepared 5-FU-MBs in various organs in mice with FaDu cell tumors were evaluated at 10 min after the IV injection of 5-FU or 5-FU-MBs with or without US (Figure 7C). At 10 min, the concentrations of 5-FU in various organs differed significantly between 5-FU-MBs + US and the other two treatment groups. Examining 5-FU levels indicated that the tumor accumulated the largest amount of 5-FU among the examined organs, followed by the kidney, liver, and then plasma. 5-FU-MBs treatment alone significantly decreased 5-FU uptake in the various organs when compared with 5-FU treatment alone, suggesting that 5-FU combined with MBs significantly reduces the 5-FU metabolism rate of the organs and extends the lifetime of 5-FU *in vivo*. The 5-FU levels in tumors were significant higher after 5-FU-MBs + US treatment than after either 5-FU-MBs (2.66 ± 0.38 vs 1.99 ± 0.12, *p* < 0.01) or 5-FU (2.66 ± 0.38 vs 2.19 ± 0.21, *p* < 0.05) treatment. The therapeutic outcome of *in vivo* low-dose 5-FU in an experimental head and neck cancer model treated with USMB and 5-FU-MB-mediated chemotherapy for 34 days could be predicted and investigated.

### 3.7. Low-dose 5-FU chemotherapy with 5-FU-MBs + US provides effective treatment for head and neck cancer

**Figure 8.**
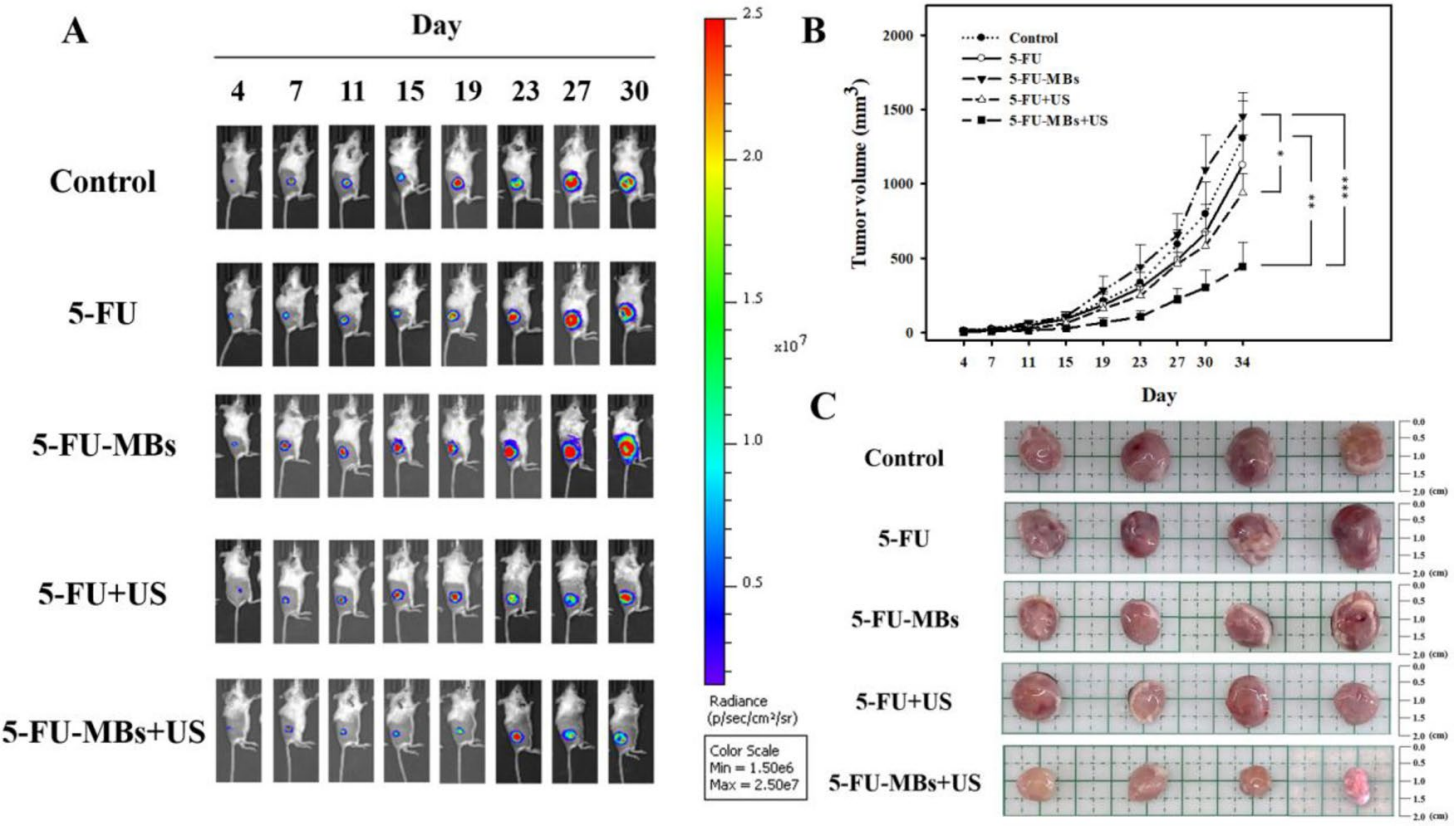
(A) *In vivo* imaging system to monitor tumor growth and chemotherapy outcomes in living mice. (B) Caliper growth curve of each xenograft experimental group during the experimental chemotherapy course. (C) Photographs of tumors in each treatment group after 34 days. Data are mean and SEM values (*n* = 5). *p < 0.01, **p < 0.01, ***p < 0.001 (two-way ANOVA with Tukey’s multiple comparison test).

Figure 8 shows the tumor sizes estimated using caliper measurements and the BLI intensities at the tumor sites. The largest tumor growth inhibition was achieved in the group that received 5-FU-MBs + US, where the tumor volume was controlled remarkably over time. Representative results shown in Figure 8(A) indicate that the other treatment groups still exhibited different degrees of tumor expansion, especially in the control, 5-FU, and 5-FU-MBs groups; necrosis was even observed in the center of tumors. At 34 days after chemotherapy, the 5-FU + US and 5-FU-MBs + US groups showed significantly suppressed tumor growth compared with the control, with 28.0% and 65.9% reductions, respectively. The tumor growth reduction in the 5-FU and 5-FU-MBs groups did not differ significantly (Tukey’s multiple comparisons test, *p* = 0.879) from the control after 34 days of treatment.

At the end of the investigation, the mice were sacrificed and the tumors were removed and weighed. As shown in Figure 8(C), mice injected with 5-FU-MBs and exposed to US sonication showed significant tumor growth inhibition and decreased tumor weights compared with the other treatment groups. Furthermore, the mean BLI intensity for each mouse/group was quantified by measuring relative BLI intensity (Supplementary Figure 1). The results indicate that 5-FU-MBs combined with US can additively enhance *in vivo* 5-FU uptake into head and neck cancer cells at a therapeutic dose of 2.5 mg/kg.

### 3.8. Histological examinations of 5-FU-based chemotherapy in tumors and the main metabolic organs

**Figure 9.**
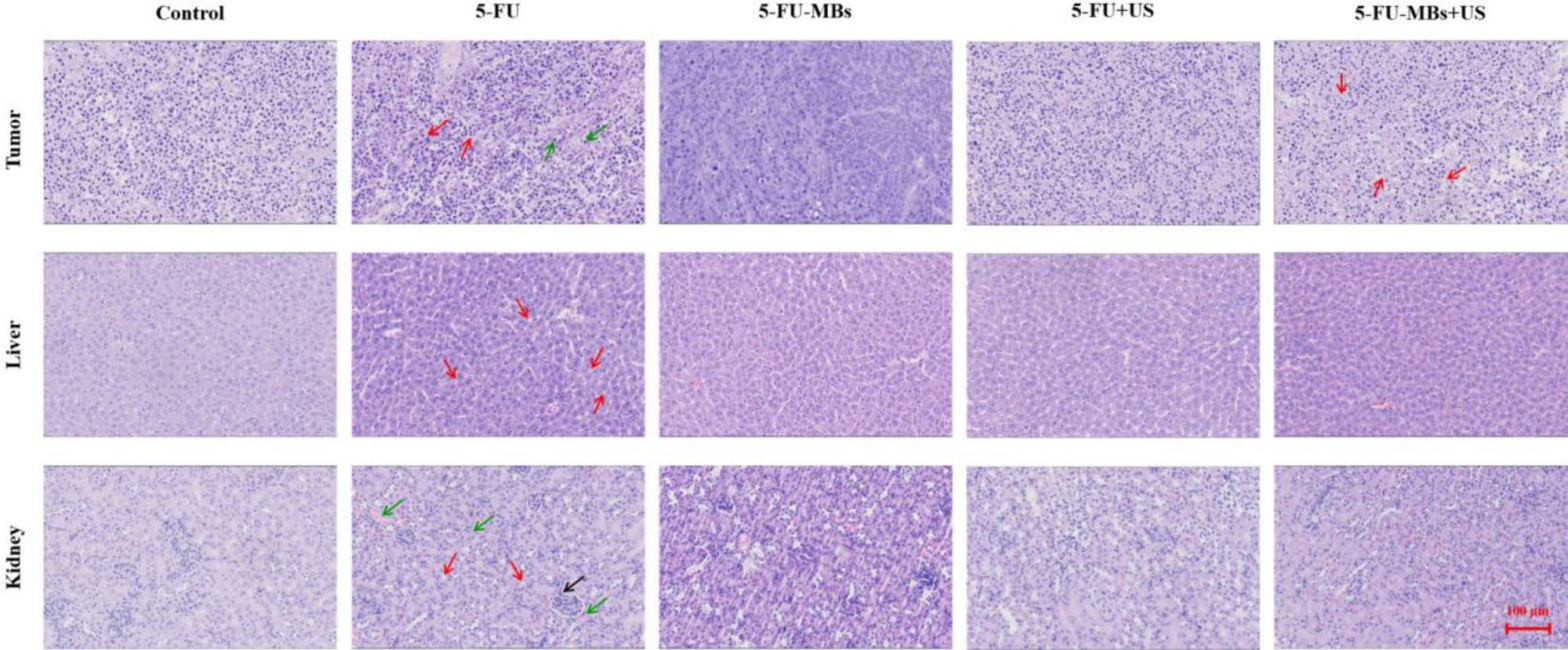
Histological examinations after 34 days of various 5-FU-based chemotherapies after removing the FaDu xenograft tumors, livers, and kidneys of SCID mice.

The light-microscope images of H&E-stained tumors (histopathological examinations) after 34 days in Figure 9 indicate that tumor cells were closely arranged with complete and atypical structures in control group. In the 5-FU chemotherapy groups, we observed greater tumor cell apoptosis, which was characterized by incomplete cell membranes (red arrows), condensed cytoplasm, nuclear condensation and nuclear fragmentation (green arrows). Vacuolated cells, intercellular edema, and multiple cyst formation (red arrows) were more commonly observed in the 5-FU-MBs + US group. The systemic cytotoxicity of 5-FU chemotherapy in the nontarget liver tissue indicated that low-dose 5-FU treatment resulted in the development of slight liver steatosis, increased hepatocellular lipid content (red arrows), and irreversible hepatocellular damage via inflammatory cell recruitment, especially in the 5-FU treatment group. Histological analysis of control renal parenchyma indicated that kidney function and architecture were normal. In contrast, administration of 5-FU alone resulted in more-obvious disruption of the normal renal architecture (red arrows) than in the other treatment groups, which was evident through blood sinusoids, interstitial hemorrhages (green arrows), glomerular congestion, atrophy, and inflammatory cell infiltration (black arrow).

## 4. Discussion

Compared with the physiological status of normal tissues, the tumor microenvironment has drastically different angiogenesis, perfusion, oxygenation, and metabolic state characteristics (Allinen et al., 2004; Whiteside, 2008). By adapting to insufficient tumor oxygen supply resulting from the heterogeneously distributed tumor vasculatures, tumor cells utilize the energy produced from oxygen-independent glycolysis for survival (Kim & Dang, 2006; Cairns et al., 2011). Acidification of the extracellular tumor microenvironment is therefore often in the pH range of 5.8–6.5 (compared with 7.2–7.4 for normal tissue), leading to increased tumor metastasis and treatment resistance (Gerweck & Seetharaman, 1996; Rohani et al., 2019). Some previous studies attempted to design different types of cancer-targeted nanotheranostics for precision cancer nanomedicine by targeting the unique acidic tumor microenvironment (Feng et al., 2018). Other research implemented pH-dependent antibodies capable of selective binding in the acidic microenvironment to improve the safety of antibody-based drugs (Zou et al., 2022). In the present study, the *in vitro* release investigation indicated that US sonication increased the release efficiency of 5-FU from 5-FU-MBs from 43.7% to 58.6% at pH 5, and from 39.9% to 51.2% at pH 7.4 after applying ultrasound energy for 2–6 hours. These results suggest that the acidic tumor microenvironment can increase the US-mediated release efficiency of 5-FU from 5-FU-MB, thereby enhancing drug release ability for tumor treatment.

The results of the *in vitro* cell viability analysis indicate that without the addition of US, the cell viability in the control group was not affected by the presence or absence of MBs, indicating that MBs would not affect cell growth. In the 5-FU + MBs and 5-FU-MBs groups using different concentrations of 5-FU without US, it was found that the apoptotic effect of 5-FU-MBs was more obvious than that of 5-FU + MBs. This may be due to the distributions of 5-FU and MB not being synchronized in the medium, resulting in poor apoptosis efficiency in the 5-FU + MBs group. It can be observed that 5-FU + MBs with a concentration of 0.5 μM without US resulted in cell viability that was close to that in the control group. At drug concentrations of 0.5–2.5 μM without US, the *in vitro* cell viability in the 5-FU-MBs group was similar to that in the 5-FU group, and significantly reduced in 5-FU-MBs groups after US, especially at higher 5-FU concentrations (1.25 and 2.5 μM). This indicates that the US-mediated MB cavitation increases cell membrane permeability, and releases the 5-FU encapsulated within MBs to increase the probability of the drug entering the tumor cells.

In the *in vivo* animal experiments, the low 5-FU dose used in the treatment resulted in the treatment efficiencies being similar in the control, 5-FU, and 5-FU-MBs groups, and there was no tumor-suppressing effect. The 5-FU-MBs + US group was found to have the strongest effect. The inertial cavitation effect generated by the US-induced 5-FU-MB destruction caused the drug to be released into the tumor microenvironment. Moreover, to determine tumor inhibition effects, the differences between more treatment conditions need to further exploration.

5-FU is an effective chemotherapeutic agent that is extensively employed in systemic chemotherapy for solid tumors such as in the head and neck, breast, colorectal, and brain cancers. In clinical practice, continuous 5-FU administration is required to maintain its therapeutic activity, but side effects may occur (Dalwadi & Patel, 2018). The present study evaluated the side effects of organ metabolism by observing biodistribution and histology. At 10 min after 5-FU or 5-FU-MBs injection, the 5-FU was mostly distributed in the kidney and tumor. Compared with 5-FU, when 5-FU-MBs were injected into animals, the 5-FU concentration decreased in all organs. At this stage, the structure of 5-FU-MBs can prolong the lifetime of 5-FU and protect various metabolic organs from being damaged by its toxicity. After US destroyed the 5-FU-MBs structure, the 5-FU concentration increased in all metabolic organs. This means that 5-FU-MBs without US cannot effectively release 5-FU into various metabolic organs. Damage and disruption of the normal renal architecture were more obvious in the 5-FU-alone group than in the other treatment groups.

The present *in vitro* and *in vivo* data indicate that the combination of 5-FU and MB does not significantly affect 5-FU cytotoxicity *in vitro*, and decreases 5-FU cytotoxicity for tumor cells *in vivo*. However, an obvious antitumor effect of 5-FU-MBs was achieved when combined with US sonication in both *in vitro* and *in vivo* experiments. Likewise, the use of the USMB technique to increase cell permeability and enhance 5-FU uptake and apoptosis would result in a better tumor reduction outcome for low-dose 5-FU treatment.

## 5. Conclusion

This study developed a new platform to improve the 5-FU limitations of short lifetime, high injection dose, and long treatment duration, and to potentially decrease tumor metastasis and treatment resistance in an acidic tumor microenvironment. US sonication significantly enhanced the release efficiency of 5-FU from 5-FU-MBs at pH 5 in the *in vitro* release study, which mimicked the acidic tumor microenvironment. Moreover, US-mediated 5-FU-MBs cavitation increased the permeability of tumor cell membranes, and released 5-FU that then entered the tumor cell. The *in vivo* results indicate that 5-FU-MBs combined with US can enhance the 5-FU uptake and inhibit the tumor growth rate of head and neck cancer cells, even at a low 5-FU dose. The new platform induces greater tumor cell apoptosis while avoiding renal and liver damage compared with 5-FU alone and other treatments.

## Supplementary Material

Supplemental Material
