# Supplementary figures and images for: Treatment efficacy of low-dose 5-fluorouracil with ultrasound in mediating 5-fluorouracil-loaded microbubble cavitation in head and neck cancer
Source: Drug Deliv. 2022 Dec 29;30(1):1–13. doi: 10.1080/10717544.2022.2154410 (PMC9809406; doi:10.1080/10717544.2022.2154410)

**Supplementary data**


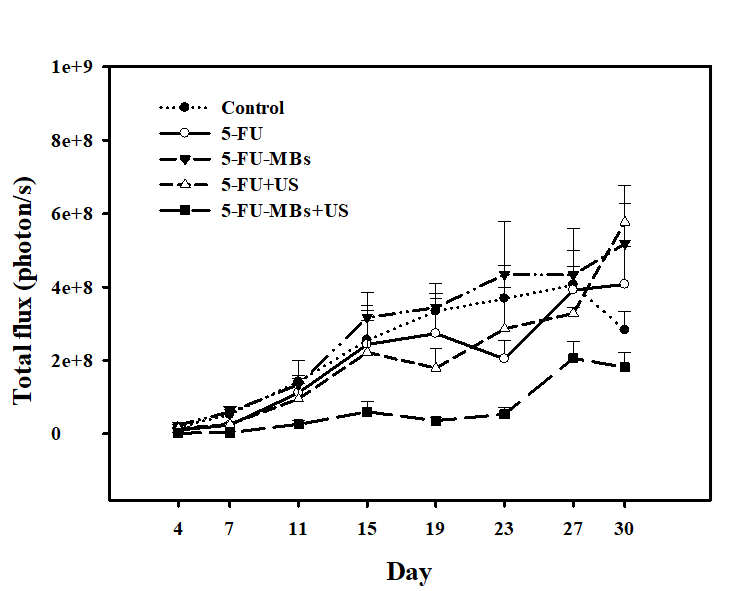


**Supplementary Fig. 1.** Analysis of the BLI images in Fig. 8A.

Supplement: Supplemental Material [file IDRD_A_2154410_SM2664.docx]
